# Effects of Extracts of Two Selected Strains of *Haematococcus pluvialis* on Adipocyte Function

**DOI:** 10.3390/life13081737

**Published:** 2023-08-13

**Authors:** Ilaria Pappalardo, Anna Santarsiero, Rosa Paola Radice, Giuseppe Martelli, Giulia Grassi, Marcos Roberto de Oliveira, Vittoria Infantino, Simona Todisco

**Affiliations:** 1 Department of Science, University of Basilicata, Viale dell’Ateneo Lucano 10, 85100 Potenza, Italy; ilaria.pappalardo@unibas.it (I.P.); anna.santarsiero@unibas.it (A.S.); rosapaolaradice@gmail.com (R.P.R.); giuseppe.martelli@unibas.it (G.M.); 2 Bioinnova Srls, Via Ponte Nove Luci, 22, 85100 Potenza, Italy; 3 School of Agriculture, University of Basilicata, Forest, Food and Environmental Sciences, Viale dell’Ateneo Lucano 10, 85100 Potenza, Italy; giulia.grassi@unimol.it; 4 Departamento de Bioquímica Rua Ramiro Barcelos, Universidade Federal do Rio Grande do Sul (UFRGS), 2600 Anexo Santa Cecília, Porto Alegre 90610-000, RS, Brazil; mrobioq@gmail.com

**Keywords:** microalgae, adipocyte, obesity, lipid metabolism, ACLY, mitochondrial function

## Abstract

Recently, microalgae are arousing considerable interest as a source of countless molecules with potential impacts in the nutraceutical and pharmaceutical fields. *Haematococcus pluvialis*, also named *Haematococcus lacustris,* is the largest producer of astaxanthin, a carotenoid exhibiting powerful health effects, including anti-lipogenic and anti-diabetic activities. This study was carried out to investigate the properties of two selected strains of *H. pluvialis* (FBR1 and FBR2) on lipid metabolism, lipolysis and adipogenesis using an in vitro obesity model. FBR1 and FBR2 showed no antiproliferative effect at the lowest concentration in 3T3-L1 adipocytes. Treatment with FBR2 extract reduced lipid deposition, detected via Oil Red O staining and the immunocontent of the adipogenic proteins PPARγ, ACLY and AMPK was revealed using Western blot analysis. Extracts from both strains induced lipolysis in vitro and reduced the secretion of interleukin-6 and tumor necrosis factor-α. Moreover, the FBR1 and FBR2 extracts improved mitochondrial function, reducing the levels of mitochondrial superoxide anion radical and increasing mitochondrial mass compared to untreated adipocytes. These findings suggest that FBR2 extract, more so than FBR1, may represent a promising strategy in overweight and obesity prevention and treatment.

## 1. Introduction

Microalgae are unicellular photosynthetic life forms that can live in various climatic conditions and aquatic habitats [1]. Among them, the most investigated are *Chromochloris zofingiensis*, *Spirulina*, *Haematococcus*, *Dunaniella* and *Scenedesmus*, considered potential model organisms for biotechnological applications like waste water bioremediation [2], biodiesel production [3] and food supplements [4]. A recent investigation analyzing a possible cost-effective application found critical limits in the form of low biomass productivity and the high costs of commercial algae growing [5].

The growing market interest for nutraceuticals and natural products forces us to ameliorate the negative aspects of their production by optimizing microalgal growth conditions and selecting microalgal strains that accumulate different metabolites at high levels, such as carotenoids. These are compounds with great beneficial effect that are largely utilized as components of dietary supplements and drugs and for cosmetic applications [6].

It should be noted that several parameters including lighting, pH, CO_2_ intake and nutrients affect the type and amount of metabolites in microalgae [7], particularly secondary carotenoids, of which accumulation is a response to different stress conditions [6].

Microalgae are used to produce countless nutrient compounds, like vitamins, minerals, polysaccharides, peptides, enzymes, sterols, polyunsaturated fatty acids and photosynthetic pigments (such as some carotenoids and chlorophylls), antioxidants, antibacterials and immunostimulants [8,9].

Their anti-obesity effect indicates a possible promising application of microalgal extracts, which could contribute to metabolic modulation. Recently, this modulation has been met with growing interest as a potential approach investigated in a variety of disease contexts for both diagnosis and treatments [10,11,12].

Obesity is a multifactorial metabolic disorder and a critical risk factor for the development of diabetes mellitus, inflammatory diseases, cardiovascular disease, hypertension, dyslipidemia, chronic inflammation and some kinds of cancer. It starts as a prolonged imbalance in energy homeostasis, the basis of the onset of overweight. Body weight gain is closely related to growth in the mass of white adipose tissue [13,14], leading to an inflammatory state. The onset of obesity is associated with chronic inflammation, which increases the production of inflammatory cytokines and mediators, alters energy homeostasis and causes dysfunctional metabolism, resulting in the associated comorbidities [15]. Adipose tissue, beyond storing a surplus of triglycerides, is recognized as a vital endocrine organ producing different adipokines (leptin, adiponectin and resistin, among others) and activating cytokines that affect metabolic homeostasis, innate immunity and insulin resistance [16,17,18]. In conditions of obesity, there is an increase in both size (hypertrophy) and number of adipocytes (fat cell hyperplasia). Adipocyte hypertrophy is closely associated with a large release of mediators like tumor necrosis factor (TNF) and interleukin-6 (IL-6) [19], which induces a boost in inflammatory infiltration into adipose tissue. Conversely, adipocytes characterized by lower lipid content are likely to release more “healthy” adipokines [20]. Moreover, adipose tissue includes different kind of cells such as preadipocytes, macrophages, other immunological cells and mature adipocytes [21]. Particularly, macrophages surround adipocytes in obese visceral fat, forming “crown-like structures” with a prevailing number of M2 macrophages. In obesity, more M1 macrophages infiltrate, together with a significant switch in the M1/M2 ratio, fostering a proinflammatory state [22]. The excessive accumulation of triglycerides induces adipogenesis, the process leading to the differentiation of preadipocytes into adipocytes. This process generates a great amount of adipocytes, which become the predominant cells and thus form adipose tissue [23]. The transcriptional regulation of critical genes including peroxisome-proliferator-activated receptors (PPARs), 5’ AMP-activated protein kinase (AMPK), and the induction of lipogenic genes such as sterol regulatory-element-binding protein (SREBP), acetyl-CoA carboxylase (ACC) and fatty acid synthase (FAS) are essential in adipogenesis [24]. An important role could also be played by ATP citrate lyase (ACLY), an enzyme at the crossroads between lipid and carbohydrate metabolism and related to the inflammatory response in macrophages [25].

Molecules or drugs regulating the expression of pivotal genes/proteins that affect adipogenesis can represent a potential approach in obesity prevention and treatment [26]. It has emerged that several phytochemicals can impact adipocyte function [27], and the literature continuously confirms the powerful health effects of microalgae. *Haematococcus pluvialis*, also named *Haematococcus lacustris* [28], is a microalga with a very varied and complete biochemical composition. It is marked by a considerable protein content, ranging from 29% to 45% (dry weight). During the green stage, the vegetative phase where the cells are green and grow by dividing themselves, i.e., a favorable phase for biomass accumulation [29], the intracellular lipid amount fluctuates between 20% and 25%, with about 10% represented by polyunsaturated fatty acids (PUFAs) [30], the richest source of astaxanthin, a natural carotenoid whose antioxidant [31], anti-inflammatory [32] and anti-diabetic [33] properties are well known.

In this study, we selected two strains of *H. lacustris/pluvialis*, named FBR1 and FBR2, and we investigated the effect of their extracts on adipocyte function.

The present study aims to contribute to elucidating the effect of *H. lacustris*/*pluvialis* extracts on the metabolism of adipocytes, highlighting a possible protective and preventive function in the development of alterations that are linked to obesity. Our attention is also focused on the mitochondrial function of adipocytes as a potential target for these extracts.

## 2. Materials and Methods

### 2.1. Microalgal Cell Culture and Growth

*H. pluvialis* UTEX 2505 (HP Wt) strain was grown in a home-made medium and conditions as reported in Radice et al. [34]. The optical density at 750 nm (SPECTROstar^®^ Nano, BMG Labtech, Ortenberg, Germany) and the number of cells via optical microscopy (Zeiss Axioplan, Thornwood, NY, USA) with a Burker counting chamber (Blaubrand^®^ Wertheim, Germany) were used to assess cell growth. The random mutagenesis procedure using UV rays was carried out by Bioinnova S.r.l (Potenza, Italy). Briefly, a glass microcapillary was used to expose 100 μL of *H. pluvialis* under UVB (315 nm) rays for 15 min to produce the two chosen genotypes of HP Wt (FBR1 and FBR2). The microalgae were then planted in a Petri dish with materials of our own production and allowed to grow for 14 days. The FBR1 and FBR2 colonies with the best biometric parameters were chosen for growth in liquid media and subjected to further analysis. The growth of mutant strains was followed as previously described [34]. At the end of the growth cycle, the cells were centrifugated at 4.500× *g* for 3 min to obtain the wet biomass. The collected biomass was subjected to a lysis process involving the use of mechanical methods, detergents and organic solvents.

### 2.2. Biochemical Composition

The total fat content was determined as described by the Soxhlet method: 150 mg of dried algae was mixed with diethyl ether to extract and determine the total percentage of fat content [35]. From the fat extracted, fatty acid methyl esters (FAMEs) were obtained via transesterification with a cold methanolic solution of potassium hydroxide. An HP 4890D gas chromatograph equipped with a split/splitless injector and a flame ionization detector, both at a temperature of 260 °C, a capillary SP2560 column (100 m, 0.25 mm, and 0.2 μm) (Supelco Inc., Bellefonte, PA, USA) and a HP3398a GC Chemstation Software, version number G2072-90020 (Hewlett Packard, USA) for data processing were used. The operating conditions were: column temperature = 45 °C (2 min) −4 °C min^−1^ to 240 °C (15 min), carrier gas = hydrogen (18 cm min^−1^), split injection ratio = 1:100. No internal standard was used. Instead, FAME identification was performed by comparison with standard certificate material, Supelco FAME 10 mix 37 (CRM47885), according to AOCS Official Method Ce 1b-89. To determine the antioxidant power of the tested extracts, a 2,2′-azinobis-(3-ethylbenzothiazoline-6-sulfonic acid) (ABTS) assay and Ferric Reducing Antioxidant Power (FRAP) assay were carried out according to the methodology described by Simonetti et al. [36], with some modifications. The ABTS assay measures the antioxidant capability of both hydrophilic and lipophilic compounds, such as flavonoids and carotenoids, whereas the FRAP assay measures the reaction between iron (II) and antioxidants with SH-groups. The results are expressed as milligrams of Trolox equivalents (TE) per gram of sample. The Velioglu et al. [37] method was modified to determine the total phenols in the microalgae biomass. Briefly, 200 μL of Folin–Ciocalteu reagent (1:10; *v/v*) was mixed with 200 μL of the extract and allowed to stand at room temperature for 3 min. The mixture was incubated with 1.5 mL (5%) sodium bicarbonate solution for 60 min at room temperature. After the incubation, the absorbance was measured at 750 nm. In order to quantify the total phenols, a calibration curve was made of standard solutions of gallic acid (25–150 μg mL^−1^) at known concentrations. The results were calculated as mg gallic acid equivalent (GAE) g^−1^ dry weight of microalgae.

### 2.3. 3T3-L1 Cell Culture and Differentiation

We cultivated 3T3-L1 preadipocytes (ATCC, Manassas, VA, USA) in the Dulbecco’s modified Eagle medium (DMEM GlutaMAX, Thermo Fisher Scientific, San Jose, CA, USA), after the addition of fetal bovine serum (FBS) (10% *v*/*v*), penicillin (100 units/mL) and streptomycin (100 μg/mL) at 37 °C, in the presence of 5% CO_2_ and in an atmosphere saturated with water. Differentiation was induced 48 h after confluence (day 2) by culture in medium integrated with 10% FBS, 1 μM dexamethasone, 0.5 mM 3-isobutyl-1-methylxanthine (IBMX) and 10 μg/mL insulin for two days. After that, the differentiated cells were grown for an additional three days in medium containing insulin (10 g/mL).

### 2.4. Cell Viability Assay

To measure cell viability, 0, 20, 50 and 100 μg/mL of HP Wt, FBR1 and FBR2 were added to 3T3-L1 adipocytes for 24, 48 and 72 h. A CellTiter-Glo^®^ 2.0 Cell Viability Assay (G9242 Promega, Madison, WI, USA) was used to measure cell proliferation, as previously reported [38]. Briefly, after 10 min of incubation at room temperature, the luminescence was measured using a microplate reader (GloMax, Promega,).

### 2.5. Quantification of Lipid Content with Oil Red O Staining

The accumulation of intracellular lipids was investigated via Oil Red O staining as reported previously [39]. In short, 3T3-L1 adipocytes were incubated with microalgal extracts (20 μg/mL) for 24 h, then fixed with 4% paraformaldehyde and finally washed with 60% isopropanol. Then, fixed cells were treated with 0.5% Oil Red O sterile solution (Sigma-Aldrich, St. Louis, MO, USA) and, after being washed with distilled H_2_O, were observed by using a Evos Floid Cell Imaging Station. The lipid droplets were solubilized by incubating the plate with 100% isopropanol for 15 min. Then, a microplate reader (GloMax) was used to measure the lipid absorption/accumulation at 490 nm.

### 2.6. In Vitro Lipolysis

The lipolysis assay was carried out in accordance with the lipolysis colorimetric assay kit’s instructions from the manufacturer (Lipolysis (3T3-L1) Colorimetric Assay Kit, MAK211, Sigma Aldrich).

### 2.7. Western Blotting

The cell lysates were subjected to immunoblot analysis as previously described [40] by using specific antibodies against PPARγ (sc-7273, Santa Cruz Biotechnology, Dallas, TX, USA), ATP citrate lyase (ab40793, Abcam Cambridge, MA, USA), AMPKα1 (sc-398861, Santa Cruz Biotechnology) or anti-β-actin antibodies (ab8227, Abcam). Horseradish peroxidase’s ECL WesternBright substrate (Advansta, Menlo Park, CA, USA) was used to detect the immunoreactions using a ChemiDoc XRS+ System (Bio-Rad Laboratories, Hercules, CA, USA). Image Lab software version 5.2.1 was used for image acquisition and densitometric analysis (Bio-Rad Laboratories).

### 2.8. IL-6 and TNFα Detection

The concentrations of secreted IL-6 and TNFα were measured via Mouse IL-6 ELISA (Cat. n° 32670069U1, ImmunoTools, Friesoythe, Germany) and Mouse TNFα ELISA (Cat.n° 32673019U1, ImmunoTools) following the instructions as outlined in Santarsiero et al. [41]. Each sample was tested in triplicate. Briefly, microplates were coated with mouse monoclonal antibody against IL-6 or TNFα. The standards and samples were incubated for 2 h at 37 °C. After washing, incubation with biotinylated anti-mouse antibodies was performed for 2 h at room temperature. After adding 100 μL of streptavidin– horseradish peroxidase, the microplates were incubated for 30 min. Tetramethylbenzidine substrate solution was used for color development. The reaction was stopped by 50 μL of 2 M of sulfuric acid. Finally, the absorbance was measured at 450 nm using a GloMax^®^ microplate reader.

### 2.9. Evaluation of Microalgal Effect on Mitochondrial Function

To assess the effect of microalgal extracts on mitochondrial function, adipocytes were stained with different fluorescent probes and the fluorescence microscope EVOS FLoid Cell Imaging Station (Thermo Fisher Scientific, 20× magnification) was used to capture fluorescence as previously described [42]. In more detail, the fluorescence was measured by using both MitoTracker^®^, Green FM (MTG, Thermo Fisher Scientific) to evaluate mitochondrial mass and the MitoSOX^TM^ mitochondrial superoxide indicator (Thermo Fisher Scientific), which is a marker of mitochondrial oxidative stress and was detected via an excitation range of 482/518 nm and emission range of 532/590 nm. Conversely, the fluorescence obtained upon staining with MitoTracker^TM^ Red CMXRos (Thermo Fisher Scientific), indicating active mitochondria, was detected via an excitation range of 586/615 nm and emission range of 626/668 nm. The intensity of the mitochondrial fluorescence was measured through image analysis using ImageJ. The images are representative of three independent experiments.

### 2.10. Statistical Analysis

The results of at least three independent experiments carried out in triplicate are presented as mean values with standard deviation (SD). One-way ANOVA was used to analyze comparisons involving more than two groups, and Dunnett’s multiple comparison tests were then performed. The figure legends provide specific information on the statistical techniques used for each experiment. Statistical significance is indicated in the figures (* *p* < 0.05, ** *p* < 0.01 and ^###,^ *** *p* < 0.001, ^#^ *vs*. preadipocytes, * *vs*. adipocytes).

## 3. Results

### 3.1. Biochemical Composition

A preliminary analysis of the major biochemical components present in the microalgal strains tested for this work was performed. In particular, the total lipid and phenolic content were determined. In Table 1, the data indicate that the FBR2 strain showed the highest total phenol and fat content when compared to the HP Wt and FBR1 strains. Considering the antioxidant power of *H. lacustris/pluvialis*, the radical scavenging capacity of extracts of all strains was evaluated by using an ABTS assay and FRAP assay.

The results highlight that FBR2 is a potential source of antioxidants and more efficient compared to HP Wt.

Moreover, when we carried out a profile of the fatty acid composition for the HP Wt, FBR1 and FBR2 extracts, we observed a high percentage of saturated (from about 22% to 26% of palmitic acid), monounsaturated (about 22–23% of oleic acid) and polyunsaturated ω3, ω6 fatty acids with the best ω3:ω6 ratio in FBR2 extract (Table 2). Therefore, FBR2 displayed a higher content of total fats than HP Wt and FBR1, although the percentage of different fatty acids was comparable in the tested strains.

### 3.2. FBR1 and FBR2 Extracts Attenuate Lipid Deposition in Adipocytes

**Figure 1:**
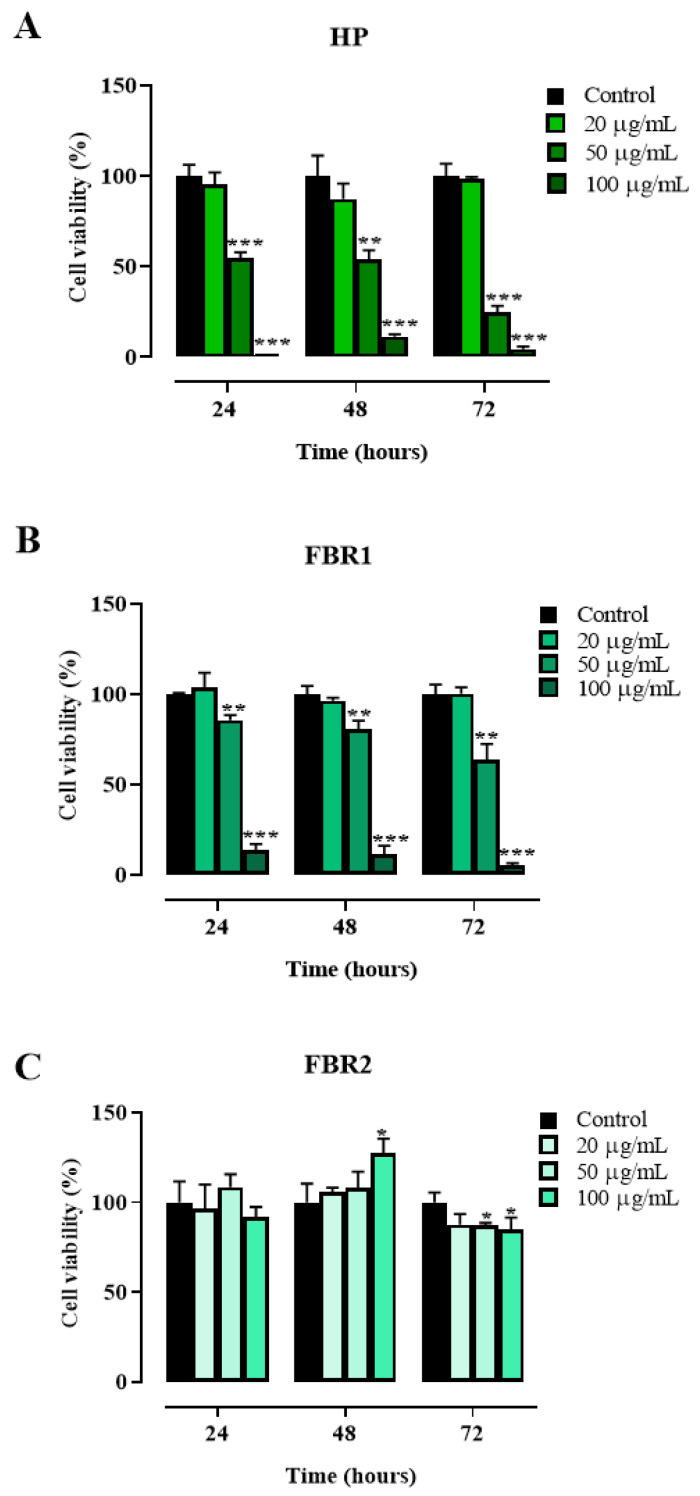
Effect of microalgal extracts on mouse 3T3-L1 adipocyte viability. (**A**–**C**). Mouse 3T3-L1 adipocytes were treated with increasing concentrations of HP Wt, FBR1 and FBR2 ranging from 20 to 100 μg/mL, and cell viability was assessed via CellTiter-Glo^®^ 2.0 Cell Viability Assay after 24, 48 and 72 h of exposure. The mean values ± SD of three independent experiments with four replicates in each are shown, where indicated differences were significant according to one-way ANOVA followed by Dunnett’s multiple comparison test (*** *p* < 0.001, ** *p* < 0.01, * *p* < 0.05).

We first examined adipocyte viability after 24, 48 and 72 h of culture in the presence or absence of 20, 50 and 100 μg/mL of HP Wt, FBR1 and FBR2 extracts. As shown in Figure 1, HP Wt had no impact on cell viability when used at the lowest concentration possible (Figure 1A). At a concentration of 100 μg/mL, FBR1 exhibited maximum toxicity, causing declines in cell number of 86%, 89%, and 95% at 24, 48, and 72 h, respectively (** *p* < 0.001, Dunnett multiple comparison test) (Figure 1B). The antiproliferative effect of FBR2 on adipocytes, on the other hand, was negligible. On the contrary, a small increase in cell proliferation was shown at 48 h in the presence of 100 μg/mL of FBR2 (Figure 1C).

**Figure 2:**
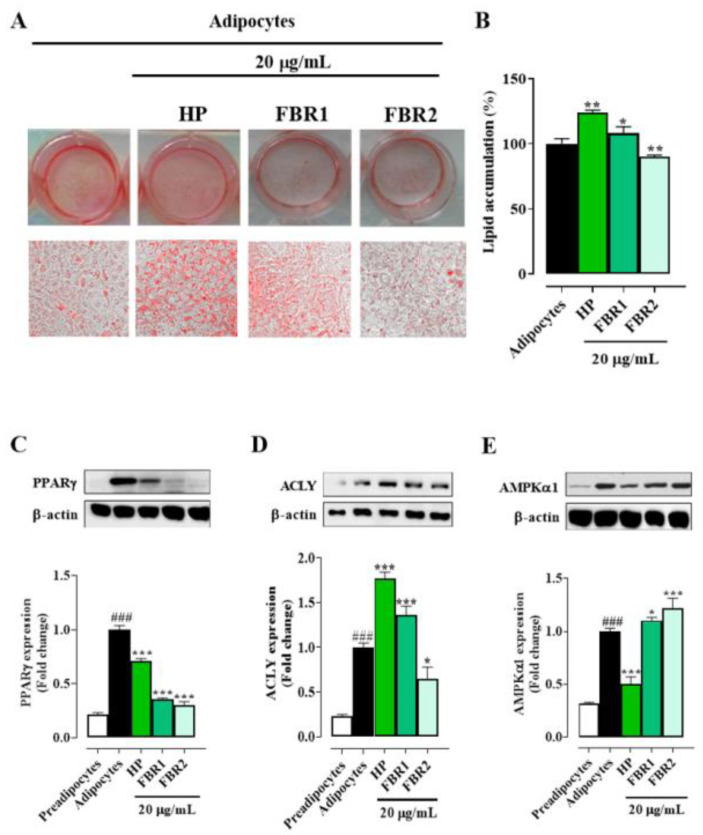
FBR1 and FBR2 extracts reduce the levels of some adipogenic markers. (**A**–**E**) Mouse 3T3-L1 adipocytes were treated with 20 μg/mL of HP Wt, FBR1 and FBR2. (**A**,**B**) Effect of microalgal extracts on lipid accumulation, determined using Oil Red O staining, in 3T3-L1 adipocytes (20× image magnification). Staining (**A**) and relative quantification (**B**) of lipid accumulation. Mean ± SD, n = 3. (**C**–**E**) Effect of microalgal extracts on adipogenic protein PPARγ (**C**), ACLY (**D**) and AMPKα1 (**E**) immunocontents. The specific bands were quantified and are presented as graphs and normalized against β-actin. The mean values ± SD of three independent experiments are shown, where indicated differences were significant according to one-way ANOVA followed by Dunnett’s multiple comparison test (^###,^ *** *p* < 0.001, ** *p* < 0.01, * *p* < 0.05, ^#^ *vs*. preadipocytes, * *vs*. adipocytes).

In light of these results, we decided to run all the subsequent experiments to assess the effect of microalgal extracts on adipocyte function and metabolism using the lowest and non-toxic concentration, 20 μg/mL. Treatment with FBR2 lowered lipid accumulation by about 10%, whereas HP Wt and FBR1 extracts increased lipid accumulation by about 25% and 10%, respectively, compared to untreated adipocytes (Figure 2A,B). As shown in Figure 2C, all tested extracts reduced the immunocontent of the differentiation-induced protein PPARγ. In particular, FBR1 and FBR2 diminished PPARγ levels by about 75% and 70%, respectively, compared to untreated adipocytes (Figure 2C). Then, we investigated if microalgae affected ACLY, a crucial enzyme in lipid metabolism. Notably, FBR2 was the extract that reduced ACLY immunocontent by about 35% compared to control cells (Figure 2D). In addition, we assessed the expression levels of AMPKα1, another target of lipid metabolism, which had an impact on lipogenesis by directly phosphorylating ACC, resulting in a decrease in lipogenic rate. Figure 2E shows that FBR2 extract increased AMPKα1 immunocontent by about 20% compared to control cells.

### 3.3. Effect of FBR1 and FBR2 Extracts on Lipolysis and Cytokine Secretion in Adipocytes

**Figure 3:**
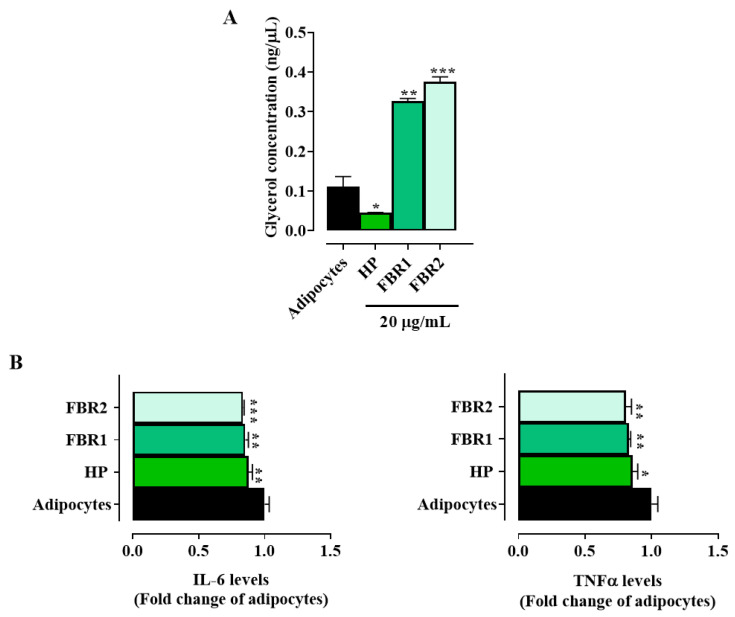
Effect of FBR1 and FBR2 extracts on lipolysis and cytokines in adipocytes. (**A**) Adipocytes were treated with HP Wt, FBR1 and FBR2 extracts. After 24 h in vitro lipolysis was assessed. (**B**) Adipocytes were treated with HP Wt, FBR1 and FBR2 extracts. Following 24 h, IL-6 and TNFα levels were evaluated and expressed as percentage of adipocytes (set at 1). Mean values ± SD of three replicate independent experiments with five replicates in each are shown. According to one-way ANOVA, differences were significant (*** *p* < 0.001, ** *p* < 0.01, * *p* < 0.05).

Treatment with FBR1 and FBR2, in contrast to HP Wt extract, significantly increased glycerol release compared to untreated adipocytes (Figure 3A), indicating a mobilization of triacylglycerol by adipocytes and a subsequent change in lipid metabolism. We then looked at how FBR1 and FBR2 extracts affected the production of inflammatory mediators, since adipocytes show an immune function. Interestingly, treatment with microalgae significantly reduced the secretion of IL-6 and TNFα cytokines, by about 20% when compared to untreated cells (Figure 3B). Altogether, the above reported data demonstrate that FBR1 and FBR2 extracts enhance lipolysis and lower proinflammatory cytokine production in adipocytes.

### 3.4. Effect of Microalgal Extracts on Mitochondrial Function

**Figure 4:**
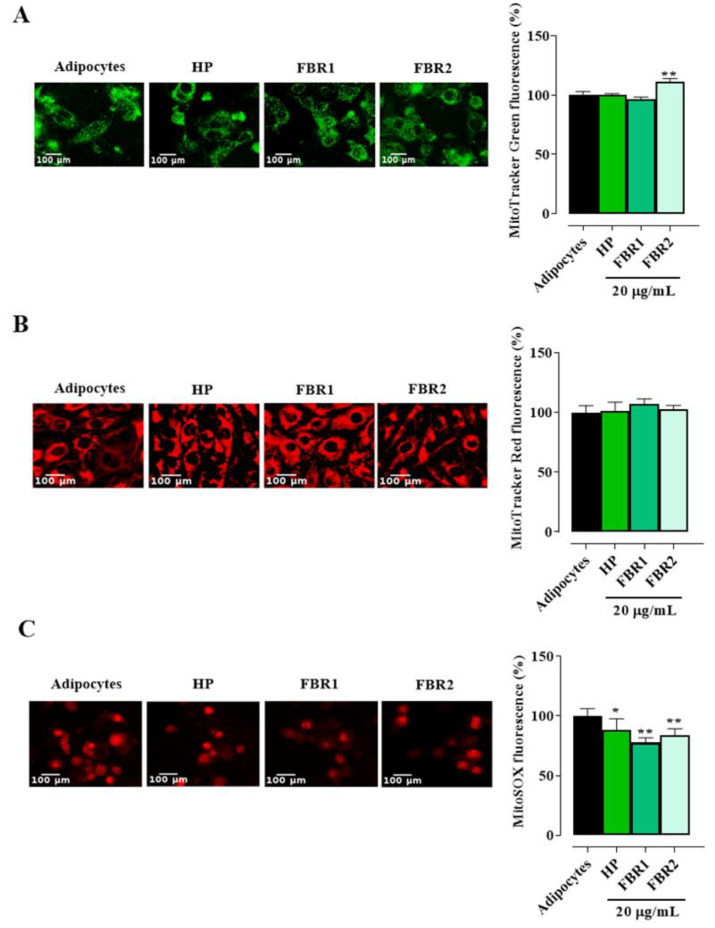
Microalgal effect on mitochondrial function. The adipocytes treated with microalgal extracts were stained with MitoTracker Green FM (**A**), MitoTracker Red CMXRos (**B**) and MitoSOX red mitochondrial superoxide indicator (**C**) and displayed under a fluorescence microscope (20× image magnification, scale bar: 100 µm). Images and related bar graphs in (**A**–**C**) are representative of three independent experiments with similar results. The mean values ± SD of three independent experiments are shown, where indicated differences were significant according to one-way ANOVA followed by Dunnett’s multiple comparison test (** *p* < 0.01, * *p* < 0.05).

Mitochondria govern several important functions and are essential for maintaining the normal physiological condition of adipose tissue. When the adipocytes were stained with MitoTracker Green, we did not observe any decrease in fluorescence intensity following treatment with microalgal extracts. FBR2 extract showed an increase of about 11% in mitochondrial mass over untreated cells (Figure 4A). No discernible differences were found between treated and untreated cells when membrane potential was examined using MitoTracker Red CMXRos (Figure 4B). On the other hand, we found a significant reduction in the production of mitochondrial superoxide anion radical following both FBR1 and FBR2 treatment (Figure 4C, ** *p* < 0.01, * *p* < 0.05, Dunnett’s multiple comparison test). These results confirm a protective effect of FBR1 as well as FBR2 extracts on mitochondrial functionality.

## 4. Discussion

Vegetable extracts represent a source of countless bioactive compounds showing a lot of biological activities [43,44,45,46]. Recently, we demonstrated the effect of red wine *Aglianico del Vulture* phenolic compounds on specific metabolic signals. Indeed, the citrate pathway, exerting pro-inflammatory activity, is downregulated upon red wine extract addition in activated macrophages. These metabolic changes are responsible for epigenetic and gene expression reprogramming, thus highlighting the potential of vegetables [47].

In this context, many studies have reported that microalgae contain a large amount of molecules with nutritional function, as well as proteins, lipids and carbohydrates [48] and health-promoting properties such as sterols, polyunsaturated fatty acids, phenolics and carotenoids, which show antioxidant, anti-inflammatory or anti-obesity effects [49]. Used as a natural food, treatment with microalgae has shown modulation of the immune system, improvement in symptoms associated with many diseases such as hypertension and fibromyalgia and the potential prevention of cancers [50]. Several microalgae, like *Euglena gracilis*, display an anti-obesity effect by regulating preadipocyte differentiation, lipid accumulation and lipogenesis [51]. These properties, affected by microalgae species, cultivation conditions and nutrient availability, are due to the different bioactive compounds identified in microalgae. Given the several healthy effects of microalgal compounds, particularly astaxanthin, among which the modulation of lipid metabolism [52], insulin resistance [53] and antioxidant [53,54], anti-inflammatory and anti-lipid peroxidation properties [30], carefully selected microalgae with high effective metabolite concentrations, rapid growth and increasing biomass could be a goal to maximize the production of bioactive compounds. Furthermore, based on the interest in anti-obesity effects, microalgal extracts with preventive anti-obesity activity open new paths towards potential new products.

In this study, starting from two new *H. lacustris/pluvialis* strains, FBR1 and FBR2, obtained by inducing high selective pressure, we demonstrated that these strains show increased biomass production, while their extracts displayed interesting biological functions compared to HP Wt. FBR2 grows faster than FBR1 and HP Wt [55], suggesting that the potential for biomass production using this novel strain should be investigated. A preliminary evaluation of biochemical composition highlights a high lipid content in FBR2 compared to HP Wt and FBR1 extracts. Recently, it has been proven that the antioxidant activity of *H. lacustris/pluvialis* extracts is related to high fat content, particularly oleic acid, and the presence of carotenoids, such as astaxanthin, which is primarily esterified with oleic acid [56].

Interestingly, a recent analysis performed to understand which compounds could be responsible for potential biological activities revealed major astaxanthin content in FBR2 (Day 0: 3.861 ± 0.501 μg/mL- Day 7: 11.568 ± 0.911 μg/mL- Day 14: 17.855 ± 0.998 μg/mL) compared to HP Wt (Day 0: 2.505 ± 0.380 μg/mL- Day 7: 8.069 ± 0.587 μg/mL- Day 14: 9.568 ± 0.432 μg/mL) and FBR1 (Day 0: 4.226 ± 0.565 μg/mL- Day 7: 8.116 ± 1.424 μg/mL- Day 14: 13.265 ± 1.642 μg/mL) [55,57].

Starting with these considerations, here we focused on the biological effects of FBR1 and FBR2 microalgal extracts specialized for the production of carotenoids. In more detail, we aimed to study their protective role in adipocyte metabolism as a form of prevention in adipose tissue health. Indeed, it is worth noting that adipose tissue lipolysis is crucial for the development of obesity [58]. Following treatment with FBR1 and FBR2, differentiated adipocytes showed a decrease in lipid deposition and lipogenesis as well as an increase in lipolysis, suggesting their involvement in metabolic changes. It has been reported that extracts of microalgae, as well as *Euglena gracilis* and *Spirulina*, significantly suppress lipogenesis in adipocytes by decreasing the expression of the key regulators of adipogenesis, PPARs and SREBP1, together with lipogenic enzymes such as ACC, and by increasing the expression of AMPKα1 [51]. FBR1 and FBR2 extracts showed a similar effect on PPARγ and AMPKα1 expression and induced a decrease in expression of ACLY, a fundamental enzyme for both lipid metabolism [59] and immune response [60]. Notably, the decrement in ACLY could be linked to decreased inflammatory mediators as shown by the reduction in IL-6 and TNFα secretion in adipocytes treated with FBR1 and FBR2 extracts. This is a very interesting outcome, as ACLY activity is closely related to cytokine gene expression and in turn production. Indeed, ACLY is the main acetyl-CoA producer, thus modulating gene expression reprogramming by epigenetic mechanisms [10,61].

The decrease in lipogenesis and the increase in lipolysis suggested for us to investigate mitochondrial function, whose impairment strongly fosters metabolic obesity-linked diseases [62,63]. Our results highlight a protective effect of FBR1 and FBR2 extracts on mitochondria because after treatment with both extracts, there was a decrease in oxidative stress, measured as superoxide anion radical, with an unchanged mitochondrial membrane potential. These outcomes appear notable when considering that high mitochondrial lipid oxidation can lead to an increase in oxidative stress. According to our findings, the presence of astaxanthin and total fats in high concentrations is the primary explanation for the protective effects of FBR1 and FBR2 extracts on adipocytes.

In conclusion, for the first time, the extracts of two new strains of *Haematococcus lacustris/pluvialis,* selected for the production of astaxanthin, were investigated for their protective effect on adipocyte function in order to evaluate their obesity prevention potential. According to our findings, FBR2, more so than FBR1 extract, may be a valuable source of anti-obesity compounds promoting lipid oxidation and inhibiting lipogenesis together with proinflammatory cytokine production.

## Figures and Tables

**Table 1:** Biochemical content of total fat, total phenolic compounds and antioxidant power evaluation of microalgal strains.

	Total Fat		Total Phenolic		ABTS		FRAP	
	%	SD	mg GAE/g lyophilisate	SD	mg TE/g lyophilisate	SD	mg TE/g lyophilisate	SD
HP Wt	16.15	1.87	17.36	0.55	35.71	1.46	46.46	1.84
FBR1	16.88	1.25	10.15	0.12	24.49	0.981	39.06	2.12
FBR2	38.40	3.07	24.61	0.47	74.00	2.25	119.67	3.13

Abbreviations: ABTS: 2,2’-azinobis-(3-ethylbenzothiazoline-6-sulfonic acid); FRAP: Ferric Reducing Antioxidant Power; GAE: gallic acid equivalents; TE: Trolox equivalents; SD: standard deviation.

**Table 2:** Composition of fatty acids in microalgal strains (%).

	HP Wt	FBR1	FBR2
C 8:0	0.11	0.11	0.09
C 10:0	nd	0.80	0.31
C 12:0	1.57	1.07	1.75
C 14:0	4.75	4.02	5.34
*cis*-C 14:1 ^Δ9^	0.84	0.59	0.88
C 15:0	1.31	1.12	1.33
*cis*-C 15:1 ^Δ10^	0.37	0.21	0.19
C 16:0	22.47	24.34	26.34
*cis*-C 16:1^Δ9^	0.73	0.64	1.06
C 17:0	2.52	1.02	1.02
*cis*-C 17:1 ^Δ10^	1.06	0.27	0.38
C 18:0	16.89	19.78	17.37
*trans*-C 18:1 ^Δ9^	0.62	0.27	0.15
*cis*-C 18:1 ^Δ9^	22.65	22.30	23.11
*cis-*C 18:2 ^Δ9,12^	10.93	11.36	8.85
C 20:0	0.44	0.91	0.66
*cis*-C 18:3 ^Δ6,9,12^	1.21	1.29	1.10
*cis*-C 20: 1 ^Δ11^	0.62	0.48	0.50
*cis-*C 18:3 ^Δ6,9,12^	2.70	2.20	2.20
C 21:0	0.36	0.30	0.20
*cis*-C 20:2 ^Δ11,14^	0.58	0.37	0.24
C 22:0	0.25	0.16	0.17
*cis*-C 20:3 ^Δ8,11,14^	0.42	0.32	0.14
*cis*-C 22:1 ^Δ13^	1.68	1.40	1.77
*cis*-C 20:3 ^Δ11,14,17^	0.51	0.22	0.26
*cis*-C 20:4 ^Δ5,8,11,14^	1.09	1.72	1.76
*cis*-C 22:2 ^Δ13,16^	0.18	0.21	0.10
C 24:0	0.07	0.09	0.01
*cis*-C 20:5 ^Δ5,8,11,14,17^	1.50	1.13	1.48
*cis*-C 24:1 ^Δ15^	0.47	0.47	0.26
*cis*-C 22:6 ^Δ3,7,10,15,16,19^	1.05	0.80	0.94

nd: not detected. Superscript numbers show the position of the double bond by counting from the carboxylic end of the molecule’s backbone.

## Data Availability

The data used to support the findings of this study are available from the corresponding authors upon request.
